# High frequency chest wall oscillation for airway clearance in bronchiectasis

**DOI:** 10.3389/fresc.2026.1890257

**Published:** 2026-07-15

**Authors:** Imad BouAkl, Jana Bazzi, Antonio Esquinas, Mohamad El-Khatib

**Affiliations:** 1 Department of Internal Medicine, Division of Pulmonary & Critical Care Medicine, American University of Beirut, Beirut, Lebanon; 2 Intensive Care Unit, Hospital Morales Meseguer, Murcia, Spain; 3 Department of Anesthesiology & Pain Medicine, American University of Beirut, Beirut, Lebanon

**Keywords:** acute exacerbation, airway clearance, bronchiectasis, high frequency chest wall oscillation, mucus, stable

## Abstract

Bronchiectasis is a chronic inflammatory airway disease characterized by impaired mucociliary clearance, persistent mucus retention, recurrent infections, and progressive airway destruction. Airway clearance techniques (ACTs) are central to the management of bronchiectasis, aiming to interrupt the cycle of mucus stasis, infection, and inflammation. High frequency chest wall oscillation (HFCWO) is a noninvasive mechanical ACT increasingly used to facilitate secretion mobilization and improve airway hygiene in patients with bronchiectasis. The objective of the current narrative review is to assess the current evidence regarding the use of HFCWO in patients with bronchiectasis, with emphasis on its physiological rationale, mechanisms of action, clinical applications, effectiveness, practical implementation, safety profile, and limitations. A narrative literature review was conducted using PubMed, Medline, Embase, Scopus, ClinicalTrials.gov, and the Cochrane Library. Relevant studies were analyzed to summarize current evidence regarding the role of HFCWO in bronchiectasis management. The evidence suggests that HFCWO may improve mucus clearance, reduce symptom burden, enhance quality of life, and decrease exacerbation frequency in selected patients with bronchiectasis. HFCWO therapy is particularly useful in patients with chronic productive cough, impaired mucus clearance, frequent exacerbations, or inadequate response to conventional ACT therapies. Practical application requires individual adjustment of treatment frequency, session duration, oscillation frequency, and pressure amplitude according to disease severity and patient tolerance. Despite its benefits, HFCWO use may be limited by high device cost, maintenance requirements, and variable long-term adherence. Reported adverse effects are generally mild and include chest discomfort, dyspnea, transient oxygen desaturation, and fatigue. HFCWO represents a valuable second-line or adjunctive airway clearance therapeutic modality and may be considered in patients with bronchiectasis, particularly those with significant secretion burden and in whom traditional and active self-administered traditional techniques have failed, are poorly tolerated, or when cognitive/physical limitations prevent active patients’ participation. Although current evidence supports its physiological and clinical benefits, future high-quality randomized studies are needed to better define patient selection criteria, long-term outcomes, adherence, and comparative effectiveness relative to other airway clearance techniques.

## 1 Introduction

Bronchiectasis is a complex and heterogenous chronic inflammatory lung disease with a wide array of infectious, autoimmune, allergic, and genetic disorders and is characterized by clinical symptoms that include breathlessness, cough, sputum production and recurrent respiratory infections (1). Some bronchiectasis cases, which are associated with different conditions such as primary ciliary dyskinesia (PCD) (2), allergic bronchopulmonary aspergillosis (ABPA) (3), non-tuberculous mycobacterial (NTM) pulmonary disease (4), and cystic fibrosis (5) have distinct guidelines for diagnosis and care. Bronchiectasis can also manifest as a secondary condition to lung diseases, such as COPD and asthma (6).

Bronchiectasis is increasingly recognized as an important chronic respiratory disease with a growing global burden. Epidemiological data suggests that both the incidence and prevalence of bronchiectasis have increased worldwide over the past decades, possibly due to greater disease awareness and advances in the use of high-resolution computed tomography (HRCT) (7). The prevalence of bronchiectasis varies substantially across populations and geographic regions, with reported estimates ranging from approximately 50 to more than 1,000 cases per 100,000 individuals in the general population (7, 8). The disease predominantly affects older adults and is more frequently observed in females, with prevalence increasing markedly with age (8, 9). Epidemiological patterns also vary geographically, reflecting potential genetic predisposition to the disease and differences in underlying risk factors such as tuberculosis prevalence, environmental and climate exposures that influence the impact of pathogens on individuals’ airways (9, 10).

The pathogenesis of bronchiectasis is complex and not yet fully understood, and it likely varies depending on the underlying etiology and several modifying factors. The airway epithelium normally serves as an important protective barrier against infection through mechanisms such as tight intercellular junctions, mucin secretion, coordinated ciliary activity, production of antimicrobial peptides, and active ion transport (10). In bronchiectasis, many of these protective mechanisms are believed to be impaired (10). It has been suggested that the disease develops in two main stages (11). An initial insult to the airway epithelium that leads to early bronchial dilation and disruption of mucociliary clearance predisposing the airways to infection, inflammation, and impaired microbial clearance leading to a second vicious stage of self-perpetuating cycle of airway injury that ultimately drives disease progression (12).

The structural airway dilation and destruction associated with bronchiectasis promote the accumulation of thick secretions, which contribute to airflow obstruction and create an environment favourable for bacterial colonization and recurrent exacerbations. Several airway clearance techniques (ACT) have been recommended to enhance mucus clearance in patients with bronchiectasis (13, 14). High frequency chest wall oscillation (HFCWO), a noninvasive and relatively independent mechanical airway clearance modality designed to enhance mucus mobilization and secretions clearance, is one of many ACTs that may be particularly relevant with significant benefits in patients with bronchiectasis (13, 14). Its ability to support regular secretion management makes HFCWO a rational adjunctive therapy aimed at reducing symptom burden, minimizing exacerbation frequency, and improving quality of life in selected patients with bronchiectasis (13, 14). The aim of the current review is to evaluate the current evidence for the use of HFCWO in patients with bronchiectasis, with particular focus on its physiological rationale, clinical effectiveness, safety profile, patient adherence, and practical application in both stable disease and acute exacerbations. The review also seeks to examine the role of HFCWO as an airway clearance modality in improving mucus clearance, reducing exacerbation frequency, enhancing quality of life, and supporting long-term respiratory management in patients with bronchiectasis.

### 1.1 Search strategy

A literature search of electronic databases including PubMed, Medline, Embase, Scopus, ClinicalTrials.gov, and Cochrane library was conducted to identify studies evaluating the use of HFCWO in patients with bronchiectasis. The search strategy was tailored to each database using a combination of controlled vocabulary terms [Medical Subject Headings (MeSH) in Medline and Emtree terms in Embase] and free-text keywords. The search focused on two main concepts: (1) high frequency chest wall oscillation and (2) bronchiectasis. Search terms related to high frequency chest wall oscillation included “high frequency chest wall oscillation”, “high frequency chest wall compressions”, “HFCWO”, “HFCWC”, and “airway clearance device”. These were combined with “bronchiectasis”. Boolean operators (i.e., “AND”, “OR”) and proximity operators appropriate for each database were applied. Searches were restricted to adult populations using database specific age filters. The search covered the period from January 2000 up to December 2025. Original articles, observational studies, registry-based studies, meta-analyses, and reviews were considered. Abstracts and studies written in a language other than English were excluded. Relevant findings were summarized narratively, focusing on the physiological rationale and evidence, clinical applications, and outcomes of high frequency chest wall oscillation in patients with bronchiectasis.

## 2 Pathophysiology of bronchiectasis as related to airway clearance

The pathophysiology of bronchiectasis is commonly described as a “vicious vortex” of impaired mucociliary clearance, chronic bacterial colonization, inflammation and airway remodeling (11). These processes are highly interdependent and a dysfunction in any one component can initiate or amplify the others, sustaining disease progression (15).

**Figure 1:**
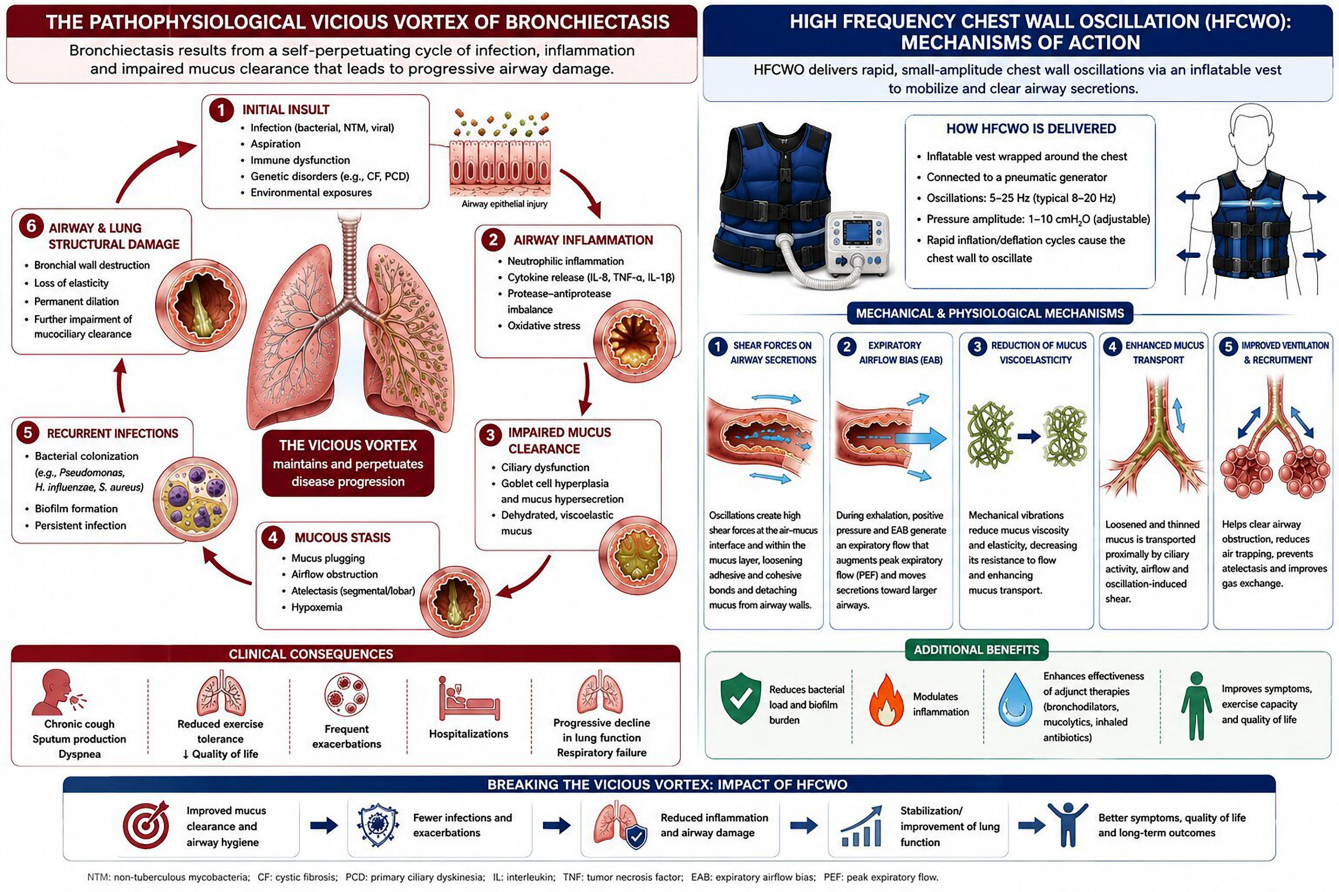
A visual aid illustration the pathophysiological vicious vortex of bronchiectasis and the mechanical mechanisms of high frequency chest wall oscillation.

Impaired mucociliary clearance leads to retention of the bronchial mucus, the disruption of the normal host defenses in cilia and the increase of airways susceptibility to chronic infections (16). Mucociliary impairment may occur due to genetic or acquired physiological and environmental factors that disrupt the normal clearance of airway mucus (17). For example, primary ciliary dyskinesia, an autosomal recessive genetic disorder, causes structural and functional defects in cilia, resulting in absent, immotile, or dyskinetic cilia (18). In addition, excessive inflammatory mediators released in response to bacterial infections, such as neutrophil elastase, can damage the extracellular matrix, reduce ciliary beat frequency, and promote mucus hypersecretion and goblet cell metaplasia (19, 20). Bacterial products, particularly from pathogens such as pseudomonas aeruginosa, may further impair mucociliary clearance by reducing ciliary beat frequency and promoting epithelial cell detachment from neighboring cells and the basement membrane (20, 21). In addition to ciliary dysfunction, mucus in bronchiectasis patients is thick and abnormally concentrated with MUC5B and MUC5AC, resulting in higher osmotic pressure and increased elasticity and viscosity (22). These abnormal viscoelastic properties impair mucus transport and may contribute to local hypoxia at the bronchial mucosa, which further promotes inflammation and mucin production (22). Together, cilia dysfunction, mucus hypersecretion, and changes in viscoelastic mucus properties render the respiratory epithelium more vulnerable to chronic infections (Figure 1).

The persistence of pathogens causative of airway infections leads to chronic airway inflammation, which is a hallmark of the bronchiectasis pathophysiology (23). Chronic airway inflammation involves a complex cytokine network that regulates the recruitment and activation of immune cells involved in host defense. This process is normally balanced by interactions between pro-inflammatory cytokines and anti-inflammatory mediators, including cytokine inhibitors that help limit the immune response (24). However, a disruption of this balance promotes persistent inflammatory cell recruitment and contributes to a self-perpetuating cycle of airway inflammation (24). The prolonged, uncontrolled protease activity accompanying the inflammatory response leads to subsequent airway tissue damage and affects lung integrity (25). In addition to the epithelial destruction, the airway obstruction associated with mucosal plugging causes structural changes to occur, including bronchial dilation and bronchial wall thickening (15, 23).

Given the central role of impaired mucociliary clearance in the development and progression of bronchiectasis, airway clearance techniques help interrupt the self-perpetuating cycle of mucus retention, infection and inflammation, leading to improved symptoms and better quality of life (13).

## 3 Overview of high frequency chest wall oscillation (HFCWO)

### 3.1 Description

High frequency chest wall oscillation, a noninvasive airway-clearance and relatively independent technique, is usually used to help mobilize and remove retained bronchial secretions. It is commonly referred to as “vest therapy” because the patient typically wears an inflatable vest or wrap around the chest which is connected to a pulse generator. The therapy involves delivering rapid (i.e.,; high frequency) inflations and deflations (i.e.,; oscillation) of the garment and thus creating repetitive external compressions of the chest wall (26). These repetitive high frequency oscillations help move mucus from the peripheral airways toward the larger central airways, where it can then be cleared by huffing, coughing, or suctioning (26).

HFCWO is a form of airway clearance therapy (ACT) that is commonly used in diseases characterized by mucus retention, such as cystic fibrosis, bronchiectasis, some chronic neuromuscular disorders, and in patients with impaired cough and excessive retained secretions. It provides independent and passive airway clearance without requiring active patient's effort or specialized breathing techniques (26).

### 3.2 Mechanism of action

The therapeutic effect of HFCWO is based on externally applied high frequency oscillatory forces transmitted through the chest wall into the bronchial tree (27). Physiologically, HFCWO works through several complementary mechanics including reduction in mucus viscoelasticity, augmentation of expiratory airflow bias, and possible enhancement of ciliary activity (27). With HFCWO, the oscillatory shear forces at the air-mucus interface help loosen secretions and make them easier to mobilize. Also, the repeated compressions generate airflow changes that help move mucus from smaller to large airways. Also, the oscillatory expiration flow generated during HFCWO can be as high as 120 L/min which is sufficient to overcome mucus adhesion from the airway wall and propel it up the airway. Finally, some evidence suggests that high frequency oscillatory airflow generated during HFCWO may improve mucociliary transport (27) (Figure 1).

In practice, HFCWO is usually paired with traditional periodic cough or huff maneuvers. It is well accepted that patients may pause HFCWO therapy every few minutes to perform forced expiratory techniques and expectorate mobilized sputum.

### 3.3 Patient interface

The main patient's interface for HFCWO therapy is an inflatable vest or wrap garment attached by tubing to an air-pulse generator. The garment/vest comes in different sizes and styles and is fitted around the thorax and sometimes upper abdomen so that oscillatory compressions are transmitted effectively to the chest wall (27). Commercial interfaces are available in different sizes including pediatric and adult options to improve fit and comfort.

Selection of the ideal HFCWO interface is usually made with attention to body habitus, age, and tolerance. Also, the interface should fit snugly enough for effective transmission of oscillations. Furthermore, the interface should remain comfortable enough for repeated use at home yet allow easy interruption of HFCWO therapy for coughing and/or suctioning.

### 3.4 Comparison with positive expiratory pressure (PEP) therapy

Positive expiratory pressure (PEP) therapy is another common airway-clearance technique. However, unlike HFCWO, PEP works through active exhalation against a resistance using a mouthpiece or mask (Table 1) (13). This creates positive pressure during expiration, which helps maintain airway patency, promotes airflow behind secretions, and may improve collateral ventilation. In oscillatory PEP devices, an additional vibration component is superimposed on the expiratory flow (28).

**Table 1:** Differences between high frequency chest wall oscillation (HFCWO) therapy and positive expiratory pressure (PEP) therapy.

Characteristic	HFCWO	PEP
Mode	Passive	Active
Transmission	Chest-wall based	Breath-dependent
Patient Criteria	Cannot reliably perform complex breathing maneuvers	Cooperative and can generate adequate expiratory effort
Equipment	High frequency pulse generator, hose, and vest/garment	Small and simple device
Cost	High	Low
Maintenance	Frequent	None

**Table 2:** Summary of studies evaluating high frequency chest wall oscillation in bronchiectasis.

Author (Year)	Study design	Population non-CF-Bronchiectasis or CF-Bronchiectasis	Sample size	Device Settings used	Primary/secondary outcomes	Main findings
Lee et al. (2017) (28)	Systematic review of randomized controlled parallel and cross-over trials	Non-CF- Bronchiectasis	9 studies; 213 participants total	Oscillatory PEP therapy (Flutter or Acapella device) and Minimal PEP therapy.	-Primary: Health-related quality of life (HRQOL), rate of acute exacerbations, and incidence of hospitalisation in individuals with stable or an acute exacerbation of bronchiectasis. -Secondary: Physiological and clinical signs and symptoms	PEP therapy had similar effects on HRQOL, sputum expectoration, lung function, and breathlessness compared to other ACTs. Oscillatory PEP (Flutter) for 4 weeks improved general health (SF−36) vs. ACBT (*p* = 0.048; 1 study). No difference in disease-specific HRQOL (CRQ total MD −0.09, 95% CI −0.37 to 0.19). No studies reported exacerbation or hospitalisation rates. Sputum yield results were mixed. One study found oscillatory PEP produced less sputum than GAD + ACBT; others found no difference. Lung function did not differ between techniques. Adverse events minimal (1 case of nausea with Flutter). Overall evidence quality: LOW (small samples, unclear blinding, short duration).
Nicolini et al. (2013) (29)	Randomized controlled trial, 3 arms [HFCWO vs. traditional chest physiotherapy (CPT) vs. medical therapy only/control], 15 days	Non-CF- Bronchiectasis	*n* = 30 (10 per arm)	1-HFCWO by using the Vest® Airway Clearance System (upright sitting position; frequency 13–15 Hz according to tolerance; pressure 2–5 cmH₂O for snug comfortable fit; 30 min/session, twice daily, 5 days/week for 15 days) 2-Traditional techniques of air way clearance (PEP bottle, PEP mask, ELTGOL, vibratory positive expiratory pressure);45 min/session, twice daily 3-Medical therapy only (control group).	-Primary: Dyspnea, cough, and sputum scales (BCSS and CAT), daily life activity evaluations. -Secondary: respiratory function testing and, hematological examinations, and sputum cell count	Traditional CPT and HFCWO showed a significant improvement in some biochemical and functional respiratory tests as well as in the quality of life compared to the control group. The use of HFCWO compared to CPT produced a significant improvement in blood inflammation parameter C-RP, parameters of lung functionality associated with bronchial obstruction (FVC, FEV1), and in the dyspnea. Improvement in quality-of-life scales was noted. No significant changes of total cell count in sputum samples were observed in the two groups. HFCWO group showed a significant reduction of neutrophils percentage and a significant increase of macrophages percentage
Clinkscale et al. (2012) (30)	Randomized trial comparing Conventional chest physical therapy (CCPT) to high-frequency chest wall compressions (HFCWC)	Mixed	*n* = 280; *n* = 146 receiving CCPT, *n* = 136 receiving HFCWC	High-frequency chest wall compressions (HFCWC): The Vest Airway Clearance System Model 205 (Hill-Rom). Frequency 10–15 Hz; up to 15 min per session followed by 5-min rest; up to 4 sessions/day. Conventional Chest Physical Therapy (CCPT): manual clapping/percussion with postural drainage; 15–20 min per session; up to 4 sessions/day. Both groups received bronchodilators prior to therapy.	-Primary: Hospital length of stay. -Secondary: ICU stay, duration of mechanical ventilation, time to radiographic resolution of lobar atelectasis, nosocomial pneumonia, hospital mortality, patient comfort (visual analog scale 1–5), adverse events.	No significant difference in hospital LOS (CCPT 12.5 ± 8.8 days vs. HFCWC 13.0 ± 8.9 days; *p* = 0.62). ICU stay, ventilator days, and 30-day mortality were similar. No nosocomial pneumonia in either group. HFCWC associated with statistically better comfort scores (1.9 ± 0.8 vs. 2.2 ± 0.8; *p* = 0.009). CCPT trended toward faster atelectasis resolution (5.2 ± 4.3 vs. 6.5 ± 5.2 days; *p* = 0.051). Trial underpowered for primary outcome. Subgroup analysis for CF/bronchiectasis showed no significant differences between groups.
Powner et al. (2019) (31)	Retrospective observational comparative cohort study (single-center treatment algorithm), 1-year pre/post comparison	Not specified	*n* = 65	Smart Vest® HFCWO system as part of a treatment algorithm (with nebulized bronchodilators, mucolytics, and macrolide therapy as indicated); Minnesota protocol, 30-min sessions, daily to twice daily	FEV1 stability, exacerbations requiring hospitalization, and antibiotic courses	FEV1 remained stable (1.85 ± 0.60 L pre vs. 1.89 ± 0.60 L post, NS). Hospitalizations for exacerbation decreased significantly (1.3 ± 1.0 pre vs. 0.46 ± 0.81 post, *p* < 0.0001). Antibiotic courses decreased modestly (2.5 vs. 2.1 per year, *p* < 0.0001). Suggests an HFCWO-centered algorithm can stabilize lung function and reduce hospitalizations/antibiotic use.
Barto et al. (2020) (32)	Retrospective registry analysis (HFCWO Outcomes Registry, Sept 2013–Nov 2015); pooled and pairwise (before/after) analyses	Non-CF- Bronchiectasis	*n* = 2,596 overall; *n* = 391 with paired pre/post data; *n* = 198 chart-review subset	High Frequency Chest Wall Oscillation Vest (HFCWO) using triangle wave percussive pulse	Respiratory hospitalizations, antibiotic use, and self-reported “overall respiratory health” and “ability to clear lungs” (5-point Likert scale), before vs. after HFCWO initiation	NCFB patients showed improved self-reported outcomes associated with the initiation of HFCWO therapy as measured by number of hospitalizations, antibiotic use, and the subjective experience of airway clearance
Urribari et al. (2024) (33)	Retrospective pre-post cohort study using commercial health insurance claims database (IQVIA PharMetrics)	Non-CF Bronchiectasis (adults; confirmed by HRCT); commercially insured US patients; mean age 55.6 years; 67% female; 58% high-risk (BACI); most common comorbidities: COPD (64%), asthma (51%)	*n* = 255 (from 4,002 who met diagnostic criteria); HFCWO prescriptions identified Jan 2009–Feb 2018	HFCWO device (HCPCS code E0483 — vest-type device); frequency and settings not specified in claims data. All patients required prior failure of standard ACT before HFCWO prescription per CMS criteria.	Primary: All-cause and disease-specific HCRU (hospitalisation, acute exacerbations, outpatient visits, antibiotic/steroid use, radiology, laboratory). Secondary: Medical costs (preindex vs. postindex 12 months).	Significant reductions 12 months post-HFCWO: disease-specific hospitalisations (*p* = 0.004); acute exacerbations (*p* = 0.007); all-cause LOS median (*p* = 0.05); oral antibiotics (*p* = 0.002); IV antibiotics (*p* = 0.01); pulmonologist visits; bronchoscopies; sputum analyses; chest radiography. Total all-cause costs similar due to device cost; disease-specific inpatient costs significantly reduced (*p* = 0.008). Excluding device cost, total costs decreased.
Sievert et al. (2016) (34)	Outcomes-based retrospective case review (pre-post design); phone interview follow-up	Non-CF Bronchiectasis (adults); confirmed diagnosis; community/outpatient setting; all compliant SmartVest users	*n* = 59 (of 104 identified; 43 excluded for non-compliance, inability to contact, death, or incoherence)	SmartVest Airway Clearance System (Electromed, Inc.); physician-prescribed regimen (frequency/duration per physician order). Used for ≥1 year.	Primary: Exacerbation-related healthcare utilisation (hospitalisation frequency, ER visits, antibiotic prescriptions, steroid use) — 1 year pre vs. 1 year post SmartVest initiation. Secondary (self-reported): Quality of life.	Significant reductions after 1-year SmartVest use: hospitalisations (*p* = 0.007); of those previously hospitalised, 65% had no readmission. ER visits (*p* = 0.083).
Sievert & Beaner (2018) (35)	Longitudinal retrospective observational case review (pre-post design); repeated phone interview follow-up over 2.5 years	Non-CF Bronchiectasis	*n* = 39 (subset of the 59 from Sievert et al. 2016 who were available for 1.5-year extended follow-up)	SmartVest Airway Clearance System (Electromed, Inc.); physician-prescribed HFCWO regimen for ≥2.5 years.	-Primary: Incidence of bronchiectasis-related exacerbations (hospitalisation admissions, ED visits, antibiotic prescriptions) — 1 year pre-treatment vs. 2.5 years post-treatment (annualised). -Secondary (self-reported): Quality of life.	Long-term SmartVest HFCWO significantly reduced bronchiectasis-related exacerbations in a non-stratified NCFB population.
Lee, Burge & Holland (2015) – Cochrane Review (37)	Systematic review and meta-analysis of randomized controlled trials (6 cross-over, 1 parallel RCT)	Non-CF- Bronchiectasis	7 studies; 105 participants total	Various airway clearance techniques (ACTs) compared to no ACT/sham/control, including HFCWO, oscillating PEP devices (Flutter, Acapella, Aerobika), ELTGOL, and postural drainage; session/treatment durations ranged from a single session to 6 months	-Primary: rate/duration of exacerbations, hospitalization incidence, and health-related quality of life (HRQoL). -Secondary: lung function, gas exchange, symptoms, sputum clearance, antibiotic use, adverse events, mortality	ACTs appear to be safe for individuals (adults and children) with stable bronchiectasis and may account for improvements in sputum expectoration, selected measures of lung function, symptoms and HRQoL. The role of these techniques in acute exacerbation of bronchiectasis is unknown.
Basavaraj et al. (2024) (39)	Retrospective observational cohort study (US Bronchiectasis and NTM Research Registry, 2008–2021)	Non-CF- Bronchiectasis	*n* = 371 with baseline HFCWO use; *n* = 103 with 1-year follow-up data (75 continued, 28 discontinued use)	High Frequency Chest Wall Oscillation (HFCWO)(duration is 20–30 min recommended twice a day)	Bronchiectasis severity by modified Bronchiectasis Severity Index and prior exacerbation between patients who continued vs. those that discontinued HFCWO at 1 year	Patients who have more severe disease and continue to experience exacerbations and hospitalizations are more likely to continue HFCWO therapy.

## 4 Clinical evidence of HFCWO in bronchiectasis

High-frequency chest wall oscillation has been evaluated in randomized clinical trials, observational studies, and registry analyses assessing its effects on symptoms, sputum clearance, lung function, exacerbations, healthcare utilization, and quality of life in patients with bronchiectasis.

Evidence from randomized clinical trials suggests that HFCWO may improve symptoms and facilitate airway clearance. In an Italian randomized controlled trial of 30 patients with confirmed bronchiectasis, participants were assigned to HFCWO using the Vest® Airway Clearance System, conventional chest physiotherapy (CPT), or medical therapy alone (29). CPT included airway-clearance techniques such as slow expiration with the glottis open in the lateral position (ELTGOL), positive expiratory pressure (PEP), and vibratory PEP devices. Both airway-clearance interventions improved symptom burden and quality of life compared with medical therapy alone, as measured by the Bronchiectasis Severity Score (BCSS), modified Medical Research Council dyspnea scale (mMRC), and COPD Assessment Test (CAT). However, HFCWO produced greater improvements in BCSS and CAT scores than CPT (*p* ≤ 0.001 for both). Pulmonary function also improved, with significant increases in FVC and FEV₁ in the HFCWO group. Sputum production increased in both airway-clearance groups, but the increase was greater with HFCWO, rising from 52.0 ± 16.9 mL to 72.5 ± 24.0 mL compared with 62.5 ± 18.9 mL to 70.0 ± 21.1 mL in the CPT group (*p* = 0.011).

A second randomized controlled trial compared the effectiveness of conventional chest physiotherapy (CCPT) with high-frequency chest wall compressions (HFCWC) delivered via a vibratory vest (30). Patients were eligible for enrollment if they had a physician's order for chest physiotherapy for conditions including lobar atelectasis, bronchiectasis, or cystic fibrosis. Among the subgroup of patients with bronchiectasis (*n* = 71), 40 patients were assigned to CCPT and 31 patients to HFCWO. Within this subgroup, the mean durations of hospital-stay, intensive care unit-stay, and ventilator days were similar between the two treatment groups, with no statistically significant differences observed. The neutral findings may reflect the heterogeneous study population, inpatient setting, and limited statistical power within the bronchiectasis subgroup.

Several observational and registry-based studies have reported reductions in exacerbations and healthcare resource utilization following HFCWO initiation. An observational comparative retrospective study conducted in the United States assessed the effectiveness of a standardized treatment protocol consisting of nebulized bronchodilators, mucolytics (hypertonic saline 3%–7% or inhaled N-acetylcysteine), and thrice-weekly macrolide therapy in patients with recurrent exacerbations, with HFCWO introduced as the final step of the treatment algorithm (31). Among the 43 eligible patients included in the study, pre- and post-algorithm data showed stability of lung function one year after implementation of the protocol, with no statistically significant changes in FEV₁, FVC, or FEF_25%–75%_. However, the number of severe exacerbations decreased significantly from 1.3 ± 1.0 hospitalizations before treatment to 0.46 ± 0.81 after initiation of the algorithm (*p* < 0.0001). The number of antibiotic courses also declined from 2.5 to 2.1 (*p* < 0.0005). The clinical relevance of this modest reduction remains uncertain. A registry-based study similarly found reductions in hospitalization and antibiotic use after HFCWO initiation (32). The proportion of patients experiencing at least one respiratory-related hospitalization decreased from 49.1% (192/391) in the year prior to treatment to 24% (94/391) in the year after starting HFCWO therapy (*p* < 0.001). Similarly, the percentage of frequent exacerbators (≥3 hospitalizations) declined from 14.3% to 5.6% (*p* < 0.001). Overall hospitalization rates decreased from 0.887 to 0.404 admissions per patient per year, representing a reduction of 54.5% (*p* < 0.001). Antibiotic use also declined significantly, decreasing from 57.7% to 29.9% after one year (*p* < 0.001). Patient-reported outcomes improved substantially, with positive responses regarding overall respiratory health increasing from 13.6% to 60.5% and perceived ability to clear lung secretions increasing from 13.9% to 76.6% after one year. These improvements were largely observed within the first month and sustained over the study period (32). Consistent with these findings, lung function improvements were also observed, with 39% and 48% of patients demonstrating at least a 4% increase in mean FEV₁ and FVC, respectively, in the year following initiation of HFCWO therapy. Similarly, a retrospective pre-post study involving 255 patients with bronchiectasis evaluated the impact of HFCWO therapy on healthcare resource utilization and medical costs (33). Twelve months after initiation of therapy, all-cause inpatient hospitalizations showed a non-significant decline of 11%. However, the median length of stay among hospitalized patients decreased from 9 to 6 days, representing a 33% reduction (*P* = 0.05). Disease-specific findings showed a significant 67% reduction in bronchiectasis-related hospitalizations (*P* = 0.004) and a 67% reduction in acute exacerbations (*P* = 0.007). In addition, the proportion of patients prescribed antibiotics declined significantly in the post-index period, with oral antibiotic use decreasing by 9% (*P* < 0.002) and intravenous antibiotic use decreasing by 47% (*P* = 0.01) (33).

Several studies have also evaluated long-term outcomes associated with HFCWO therapy. In a study assessing the SmartVest® Airway Clearance System in 59 patients with non-cystic fibrosis bronchiectasis, the number of patients hospitalized for bronchiectasis-related exacerbations decreased from 29% in the year prior to therapy to 17% in the year following initiation of treatment (*P* = 0.007) (34). Emergency department visits declined by 63% (from 8% to 3%), although this reduction did not reach statistical significance (*p* = 0.083). Antibiotic use also decreased substantially, with the proportion of patients receiving antibiotics declining from 74% to 44% (*P* < 0.001). The number of antibiotic prescriptions per subject decreased from 1.9 to 1.3, and overall prescriptions per study population decreased from 1.4 to 0.6. Steroid use also declined significantly (19% vs. 3%, *P* = 0.002). Additionally, although not formally assessed in the study protocol, 68% of participants reported an improvement in quality of life during follow-up interviews (34). Another study examining long-term use of HFCWO therapy in 39 patients with non-cystic fibrosis bronchiectasis reported similar findings (35). After 2.5 years of therapy, hospitalization rates decreased by 42% (*P* = 0.007), emergency department visits declined by 75% (*P* = 0.0002), and antibiotic use decreased by 38% (*P* = 0.007). In addition, 68% of participants reported improvements in quality of life and reductions in the frequency and severity of exacerbations (35).

## 5 Indications for high frequency chest oscillation and patient selection

HFCWO is indicated as an airway clearance modality for patients with chronic secretion retention and impaired airway clearance mechanisms, particularly when mucus burden contributes to ongoing respiratory symptoms, functional limitation, and disease progression. It is mostly indicated in patients with cystic fibrosis, non-cystic fibrosis bronchiectasis, and some selected cases of chronic obstructive pulmonary disease (COPD) or neuromuscular disorders, particularly when cough effectiveness is reduced or mucociliary clearance is compromised (36).

Patient selection should prioritize individuals with a daily productive cough reflecting a persistent mucus load requiring regular clearance, as well as patients experiencing frequent exacerbations (usually more than 2–3 events per year), where inadequate secretion clearance is a contributing factor (14). Also, HFCWO is particularly appropriate for patients with a poor response or intolerance to conventional airway clearance techniques such as physiotherapy, postural drainage, or positive expiratory pressure devices or who are unable to perform these techniques effectively due to fatigue, physical limitations, or lack of caregiver support. Additionally, HFCWO is appropriate for patients requiring a consistent, independent, and home-based therapy of airway clearance for patients who would otherwise require a caregiver to perform manual and passive techniques but it holds no advantage regarding caregiver independence over traditional self-administered devices like PEP. Although its passive nature may facilitate treatment delivery, reduce to some extent dependence on caregivers or specialized breathing techniques, and promote short-term independence and adherence (14), the bulky equipment, noise, and treatment duration of HFCWO often hinder long-term compliance compared to simpler, highly portable devices. Furthermore, careful screening is necessary to exclude from HFCWO therapy those patients with unstable chest wall injuries, active significant hemoptysis, or severe hemodynamic instability. Overall, HFCWO remains best targeted to patients with clinically significant mucus retention with symptomatic burden and suboptimal outcomes with standard therapy, as it can reduce exacerbation frequency and improve airways hygiene (32). Barto et al. reported that the number of bronchiectasis patients who had at least one respiratory-related hospitalization in the year before decreased by more than 50% compared to the year after starting HFCWO therapy while at the same time a similar decrease was observed in the number of patients who required three or more hospitalizations (32). Furthermore, they showed the number of patients who subjectively rated their ability to clear their lungs as good to excellent increased from 13.9% to 76.6% (32). Similar findings were described by Urribarri et al. who reported a greater than 50% decrease in hospitalizations of patients with bronchiectasis after 12 months of initiation of HFCWO therapy (33).

## 6 Practical considerations for HFCWO

The application of HFCWO therapy should be individualized based on disease severity, secretion burden, and patient tolerance. In stable conditions, HFCWO is typically used as a maintenance therapy to prevent secretion accumulation and reduce exacerbations. Patients usually receive 1–2 sessions per day, each lasting 20–30 min (37). This is often combined with other airway clearance strategies such as exercise, breathing techniques, or positive expiratory pressure therapy (Table 3).

**Table 3:** General settings of HFCWO for patients during stable and acute exacerbation of bronchiectasis.

HFCWO parameters	Stable condition	Exacerbation	Remarks
Setting	Home use preferred	Hospital or Home	Depends on severity
Sessions Frequency	1–2 sessions/day	Individualized	Increase based on secretion load
Session Duration	20–30 min	20–30 min (may extend)	Maintain tolerability
Clinical Goal	Maintain airway clearance	Secretion mobilization	Prevent vs. Treat
HFCWO Frequency	8–15 Hz	8–15 Hz	Range: 5–20 Hz
HFCWO Pressure	Moderate, patient-tolerated	May increase cautiously	Ensure visible chest vibration
Adjunct Therapies	Exercise, breathing techniques	Bronchodilators, mucolytics	Always combined approach with consideration of pre-medication with SABA before commencing HFCWO session
Monitoring	Symptoms, adherence	SpO2, sputum, Tolerance	Adjust based on response

SABA, short-acting bet-2 agonist; Hz, hertz; HFCWO, high frequency chest wall oscillation.

During acute exacerbations of bronchiectasis, HFCWO therapy is typically intensified to enhance secretion mobilization, reduce airway obstruction, improve airway patency, and prevent atelectasis (Table 3). The frequency and duration of HFCWO sessions should be individualized according to symptom severity, secretion burden, and patient tolerance. HFCWO is commonly incorporated into a comprehensive airway-clearance regimen that may include bronchodilators, mucolytics, and assisted coughing techniques (i.e., cough and huff) to optimize secretion clearance (13, 38)

HFCWO can be safely used in both home and institutional settings. Home use is advantageous for long-term management due to ease of use and improved adherence, while hospital use is indicated for acute care, particularly in patients with increased secretion burden or limited ability to clear secretions independently. In patients with confirmed bronchiectasis and who were compliant with their prescribed HFCWO therapy, Sievert and Beaner reported a 42% decrease in the incidence of hospitalizations, a 75% decrease in emergency department visits, a 38% decrease in antibiotic prescriptions at 2.5 years after starting HFCWO treatment (35). Furthermore, 68% of patients reported significant improvement in their quality of life and a reduction in the severity of their exacerbations (35). During hospital use with acute exacerbation, HFCWO requires monitoring of hemodynamics and oxygenation and is often combined with suctioning and noninvasive ventilation.

Device settings should be carefully titrated depending on the severity of the disease with consideration to patient's tolerance (39). Basavaraj et al. reported that patients who have more severe disease and continue to experience exacerbations and hospitalizations are more likely to continue HFCWO therapy (39). Typical oscillation frequencies range from 5 to 20 Hz, with common clinical use between 8 and 15 Hz. Lower frequencies are most efficient in mobilizing proximal secretions while higher frequencies are efficient in mobilizing distal secretion (32). Varying frequencies during a session and incorporating periodic coughing or suctioning improves effectiveness. Pressure amplitude (usually in the range of 5–20 cmH2O) should be adjusted to achieve effective chest wall oscillation while maintaining patient comfort and avoiding adverse effects. It is advisable to initiate therapy at low-moderate pressure amplitudes (i.e., 5–10 cmH2O) and gradually increase the pressure based on patient's tolerance (32). Settings of frequency and pressure amplitude should be enough to generate visible chest wall oscillation while avoiding pain, hemodynamic compromise, and/or oxygen saturation (32).

## 7 Contraindications and limitations

HFCWO is generally considered safe in patients with bronchiectasis. However, its wide range use is constrained by several contraindications, limitations, and practical challenges (14, 40). Absolute contraindications include conditions in which external chest wall oscillation may pose harm, such as in patients with unstable thoracic cage (e.g., fractured ribs or flail chest), recent thoracic or abdominal surgery, untreated pneumothorax, and active or massive hemoptysis (14, 40). Relative contraindications include severe osteoporosis, hemodynamic instability, and elevated intracranial pressure (14, 40).

Although HFCWO is typically well tolerated, reported adverse effects include chest discomfort, musculoskeletal pain, transient oxygen desaturation, dyspnea, and fatigue, particularly when higher oscillatory frequencies and/or pressures are applied without appropriate titration (29). In some patients, especially those with history of airway hyperreactivity (e.g., bronchiectasis-COPD/asthma overlap), oscillatory therapy may provoke bronchospasm, necessitating pre-treatment with bronchodilators such as short-acting beta-2 agonists (SABA) prior to commencing the HFCWO session.

Despite its efficacy in enhancing mucus clearance, HFCWO is limited by its high cost and maintenance requirements, which may reduce accessibility and limit its adoption compared to lower-cost airway clearance devices such as PEP or active cycle of breathing techniques (28, 37). Furthermore, long-term adherence remains a significant concern, as HFCWO therapy is time-intensive and may be perceived as burdensome, particularly in patients with milder disease or low symptom burden. Evidence suggests that no single airway clearance modality is universally superior, and patient preference and tolerability are critical determinants of sustained adherence and clinical effectiveness (14, 28, 37).

Successful implementation of HFCWO extends beyond device prescription and requires active involvement of the multidisciplinary rehabilitation team consisting normally of respiratory therapists/physiotherapist and/or specialized nurses. These team members play a central role in patient education, training and ongoing follow-up, ensuring correct device application and integration of HFCWO therapy into individualized airway-clearance protocols. Also, these professionals are instrumental in identifying and addressing barriers to adherence, including treatment burden, technical difficulties, and patients' misconception regarding therapy. Furthermore, they can get involved in the management of mild adverse effects, such as chest discomfort, musculoskeletal pain, fatigue, and transient dyspnea, through individualized adjustment of treatment parameters and reinforcement of appropriate and optimal implementation of the treatment protocol. Consequently, multidisciplinary support is an important determinant of long-term adherence and clinical effectiveness of HFCWO therapy.

## 8 Conclusion

HFCWO represents an important adjunctive airway clearance modality in the management of patients with bronchiectasis, particularly those with chronic mucus retention, frequent exacerbations, and may be considered primarily when traditional, active self-administered techniques have failed, are poorly tolerated, or when cognitive/physical limitations prevent active patients’ participation. By enhancing mucus mobilization and airway hygiene, HFCWO may improve symptom control, quality of life and secretion clearance. However, its use remains limited by cost, adherence challenges, and the lack of standardized treatment protocols. Further high-quality studies are needed to better define optimal patient selection, treatment settings, and long-term clinical outcomes.
